# Laryngeal mask airway facilitates a safe and smooth emergence from anesthesia in patients undergoing craniotomy: a prospective randomized controlled study

**DOI:** 10.1186/s12871-023-01972-x

**Published:** 2023-01-17

**Authors:** Cheng-Fong Wei, Yung-Tai Chung

**Affiliations:** Deparment of Anesthesiology, Chang Gung Memorial Hospital, Linkou Medical Center, No. 5, Fushin St. Kweishan District, 33305 Taoyuan City, Taiwan

**Keywords:** Deep extubation, Laryngeal mask airway, Craniotomy, Respiratory complications

## Abstract

**Background:**

Endotracheal extubation under deep anesthesia (deep extubation) has been proved to present stable hemodynamics and steady intracranial pressure during emergence from anesthesia in patients undergoing craniotomy. This study aims to examine, in comparison with deep extubation, if a laryngeal mask airway (LMA) could provide a safer and smoother emergence from anesthesia in patients undergoing craniotomy.

**Methods:**

This prospective randomized controlled study was conducted on patients undergoing elective craniotomy for brain tumors. After the complement of the surgical procedure, the patients had anesthesia maintained with end-tidal sevoflurane concentration 2.5% and also fully regained muscle power (Time Zero), they were randomly assigned to ETT Group (*n* = 29) for deep extubation or to LMA Group (*n* = 29), where the endotracheal tube was replaced by a laryngeal mask airway. The primary outcomes were respiratory complications, airway interventions and hemodynamic changes through emergence from anesthesia till 30 min following Time Zero. The secondary outcomes were re-operation incidence in 24 h, stay time in the intensive care unit and postoperative hospital days.

**Results:**

At 5 min before Time Zero either oxygen partial pressures (PaO_2_) or carbon dioxide partial pressures (PaCO_2_) between the two groups were comparable. No significant PaCO_2_ change was noted in both groups in 5 min after Time Zero, yet there was a remarkably lower PaO_2_ in ETT Group at that time point, 188.9 (± 71.1) in ETT Group vs 264.4 (± 85.4) in LMA Group. In ETT Group, coughs and snores were considerably more frequent, and thus more interventions were needed to maintain adequate respiration. From Time Zero on, blood pressures (systolic, diastolic and mean) and heart rates in ETT Group were generally higher than those in LMA Group, but the differences were insignificant at all time points except heart rate at 10 min after Time Zero. The secondary outcomes between the two groups were similar.

**Conclusions:**

Compared with deep extubation, a LMA, as a temporary airway replacement, facilitates a safer and smoother emergence from anesthesia for patients undergoing craniotomy, in terms of better oxygen saturation, fewer respiratory complications and fewer airway interventions.

**Trial registration:**

The study was conducted after receiving approval from Institutional Review Board of Chang Gung Memorial Hospital, Linkou Branch, Taiwan (registration number 202102115A3; January 27, 2022), and the clinicaltrials.gov (NCT05253404) on 23 February 2022.

**Supplementary Information:**

The online version contains supplementary material available at 10.1186/s12871-023-01972-x.

## Background

Among patients undergoing craniotomy, coughing and aching would trigger not only cardiorespiratory consequences, such as laryngospasm, upper airway obstruction, pulmonary edema, hypertension and tachycardia [1, 2], but also increase intracranial pressure. Therefore, as anesthesia providers, we would aggressively administer sedatives, opioids or anti-hypertensives to provide those patients a smooth and calm emergence from anesthesia [3]. If possible, we would remove the endotracheal tube as early as possible in this patient group, which is to prevent them from developing ventilator-associated complications in the later intensive care [4]. Endotracheal extubation performed on the patients under deep anesthesia and with fully recovered muscle power (deep extubation) has been applied in eye surgeries and craniotomies [5]. Compared with traditional awake extubation, deep extubation can eliminate airway irritation and bears less cough incidence [6], but it has also been reported to lead to more airway obstructions [7]. The placement of a LMA following endotracheal extubation during emergence from anesthesia, known as Bailey Maneuver [8], has been proposed to provide patients a patent and inirritable airway. And the LMA will be removed smoothly once patients regain consciousness. The strengths of Bailey Maneuver have been proved in thoracic [9] and neurological surgeries [10, 11], where the airway devices were removed while the patients were awake. It may also benefit smokers, asthmatics, and patients with other irritable airways [12–14]. The aim of this trial was to study if Bailey Maneuver, in comparison with deep extubation, was able to provide a safer and smoother emergence from anesthesia in patients undergoing craniotomy.

## Methods

This prospective randomized controlled trial was approved by Institutional Review Board of Chang Gung Memorial Hospital (file number 202102115A3) and registered at clinicaltrials.org (NCT05253404) on 23 February 2022. A data analysis and statistics plans were written and posted on ClinicalTrials.gov.

**Fig. 1:**
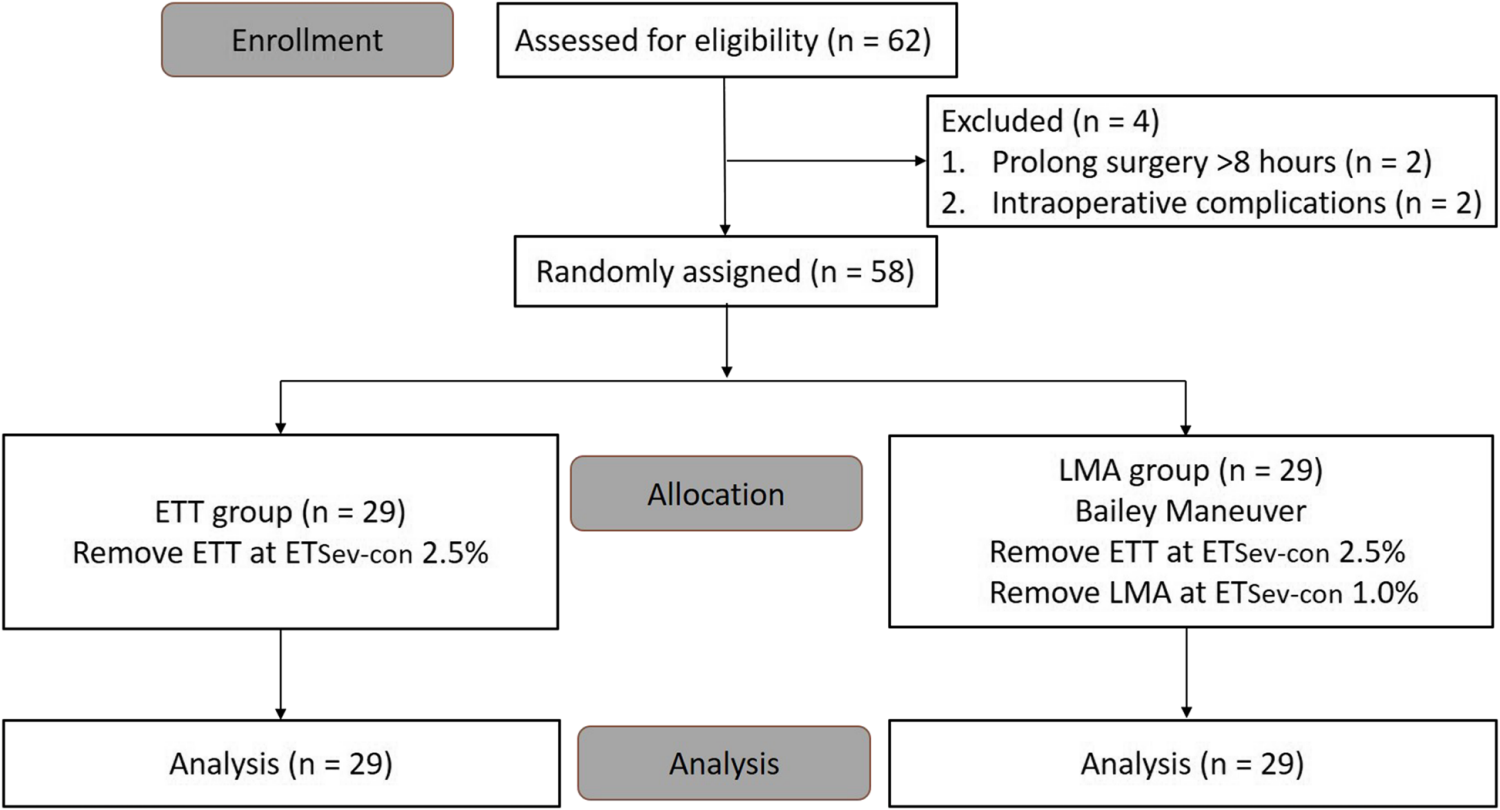
Consort flowchart. ETT, endotracheal tube; LMA, laryngeal mask airway; ETSev-con, End-tidal sevoflurane concentration

All of 62 patients, aging 20 to 65 years old and American Society of Anesthesiologists’ physical status I to III, undergoing elective craniotomy for brain tumors were recruited from 14 February 2022 until 27 June 2022. They all gave their written informed consents before anesthesia. The exclusion criteria were: anticipated difficult airway and difficult laryngeal mask airway use (mouth opening < 3 cm, Mallampati score > 3, body mass index > 30 kg/m^2^), intraoperative complications (massive blood transfusion, operative time more than 8 h) and contraindications for intubating a Laryngeal mask airway (LMA) (fasting time < 8 h). We used a computer randomization table (www.randomizer.org) to decide the assignment of the patients (Fig. 1).

Standard anesthesia working station (Carestation 620) consists of electrocardiography, pulse oximetry and muscle power monitor (response to train-of-four stimulation).

Following 3 min’ preoxygenation, all patients took induction of anesthesia with fentanyl 1–1.5 mcg/kg, propofol 1.5–2 mg/kg and cisatracurium 0.15–0.2 mg/kg or rocuronium 0.6–1.2 mg/kg injected intravenously. Oral endotracheal intubation was performed using the Clarus Video System (Trachway) with an endotracheal tube (Henan Tuoren) ID 6.5 mm -7.5 mm. Afterwards, an arterial catheter and a central venous line would be set up for all of them. Anesthesia was maintained at end-tidal sevoflurane concentration (ETSev-con) 2.0–2.5% and cisatracurium 0.03 mg/kg repeated every 40–60 min to maintain a muscle response between T1 and T2 on the train-of-four monitor. Ventilator was set to keep end-tidal carbon dioxide (ETCO_2_) between 30 and 40 mmHg.

Once surgery was completed, oropharyngeal cavity was thoroughly suctioned and the patients were prepared for endotracheal extubation. As the patients had ETSev-con 2.5% and also fully regained muscle power with continuous spontaneous ventilation (Time Zero), they were randomly assigned to ETT Group (*n* = 29) for deep extubation or to LMA Group (*n* = 29), where the endotracheal tube was replaced by a LMA the way described as Bailey Maneuver. After endotracheal extubation, the patients in ETT Group wore a face mask sitting tight on the face, and those in LMA Group would have a LMA (AuraOnce) of suitable size as the breathing tool. All of the patients breathed 100% oxygen of 6L/min during emergence from anesthesia. In LMA Group, the LMA was removed as ETSev-con arrived at 1.0% [15, 16].

The primary purpose of the study was to examine PaO_2_ and PaCO_2_ changes, respiratory complications, airway interventions and hemodynamic changes through emergence from anesthesia till 30 min following Time Zero. The study results indicate respiratory complications including coughs, snores, oxygen desaturation (PaO_2_ < 90%), aspiration, bronchospasm and pulmonary edema. In addition, airway interventions to maintain adequate respiration include chin lifting, jaw thrusting and the use of a nasal airway. The secondary purpose of the study was to examine re-operation incidence in 24 h, stay time in the intensive care unit and postoperative hospital days.

### Statistical analysis

As type I error of 5%, power of 80% (two-tailed testing) and 95% confidence intervals (CI) were used to calculate the sample size, we needed 30 participants for each group to detect the difference of mean PaO_2_. Mann–Whitney U-test was to compare ordinal data and Chi-square and fisher exact test were to find the association of respiratory complications with managements of airway. Longitudinal models, constructed by applying the generalized estimating equation methodology, were used to analyze the effect of the airway interventions on hemodynamic changes. Bonferroni correction of *p*-values was to adjust for multiplicity in time-by-time analyses. All analyses were made with SPSS version 24.

## Results

A total 58 patients were included and randomized in the study. There is no significant difference in demographic data between the two groups, except more males than females in ETT group (Table 1).

**Table 1:** Demographics of the study patients

	**ETT Group (*n* = 29)**	**LMA Group (*n* = 29)**	***p*-value**
**General**			
Sex(Male/Female)	19/10	15/14	
Age(years)	50.4 ± 10.4	47.0 ± 12.0	0.33
Height(cm)	163.0 ± 7.7	164.7 ± 10.4	0.49
Weight(cm)	63.5 ± 12.9	64.3 ± 12.2	0.70
BMI(kg/m^2^)	23.7 ± 3.7	23.7 ± 3.0	0.88
ASA score(2/3)	3/26	3/26	
Mallampati score(1/2/3)	9/19/1	12/17/0	
Glasgow Coma Scale before operative(11/12/13/14/15)	3/0/0/4/22	2/1/0/1/25	
**Surgical**			
Surgery times(minutes)	310.9 ± 110.9	328.3 ± 129.0	0.59
Intra-operative blood loss(mL)	415 ± 493	572 ± 740	0.63

Data are mean ± SD, or *n* number of patients, *BMI* body mass index, *ASA* American Society of Anesthesiologists

**Fig. 2:**
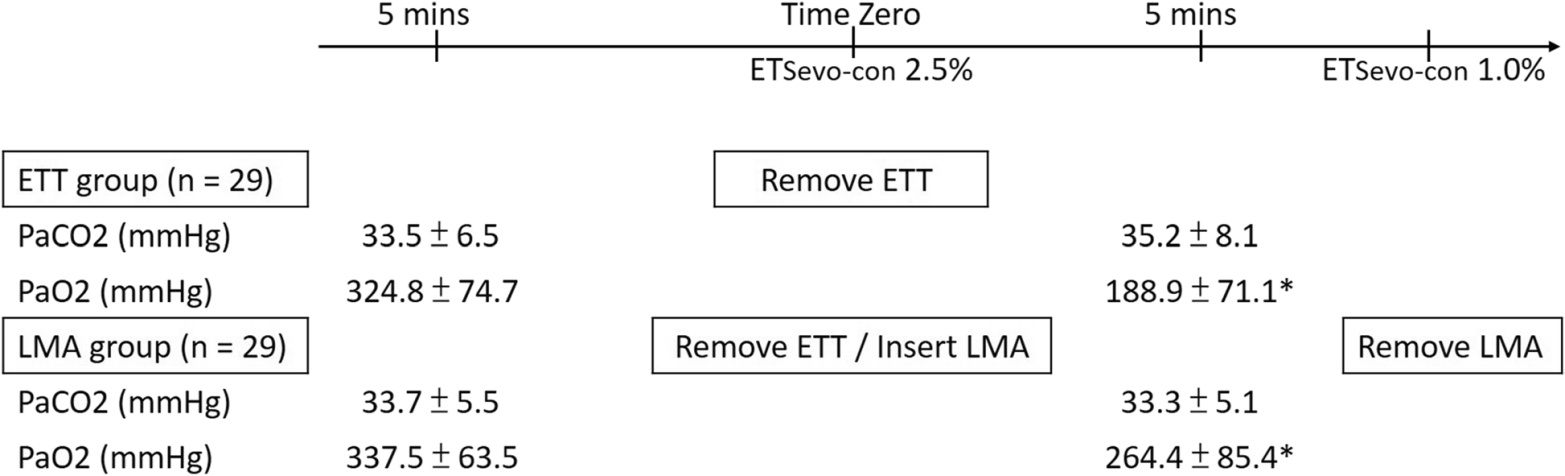
Time line of emergence from anesthesia. Data are mean ± SD, difference of means (95% CI). *p < 0.01 between two groups. ETSevo-con, End-tidal sevoflurane concentration

Numerical values of arterial blood gases suggested that the difference in PaCO_2_ and PaO_2_ between the two groups at 5 min before Time zero is nominal. The PaCO_2_ did not change significantly in either group at 5 min after Time Zero, but a considerable difference in PaO_2_ between the two groups appeared at that time point (*p* < 0.01) (Fig. 2).

Concerning respiratory complications, coughs and snores occurred significantly more frequently in ETT Group (*p* < 0.01). Oxygen desaturation was noted in 3 cases and pulmonary edema in one case in ETT Group. More patients in ETT Group needed airway interventions to maintain adequate respiration (*p* < 0.05) (Table 2); only one patient in LMA group needed a nasal airway and chin lifting after the LMA was removed.

**Table 2:** Respiratory complications in 30 min following Time Zero

Outcome	ETT group (*n* = 29)	LMA group (*n* = 29)	*p*-value
**Respiratory complication**			
Snore	21	6	< 0.01
Cough	12	1	< 0.01
Oxygen desaturation (SpO_2_ < 90%)	3	0	0.24^#^
Pulmonary edema	1	0	1.00^#^
**Airway intervention**			
Nasal airway	25	1	< 0.01
Chin lifting	19	1	< 0.01
Jaw thrusting	6	0	< 0.05^#^

Data are number of patients, ^#^fisher exact test

**Fig. 3:**
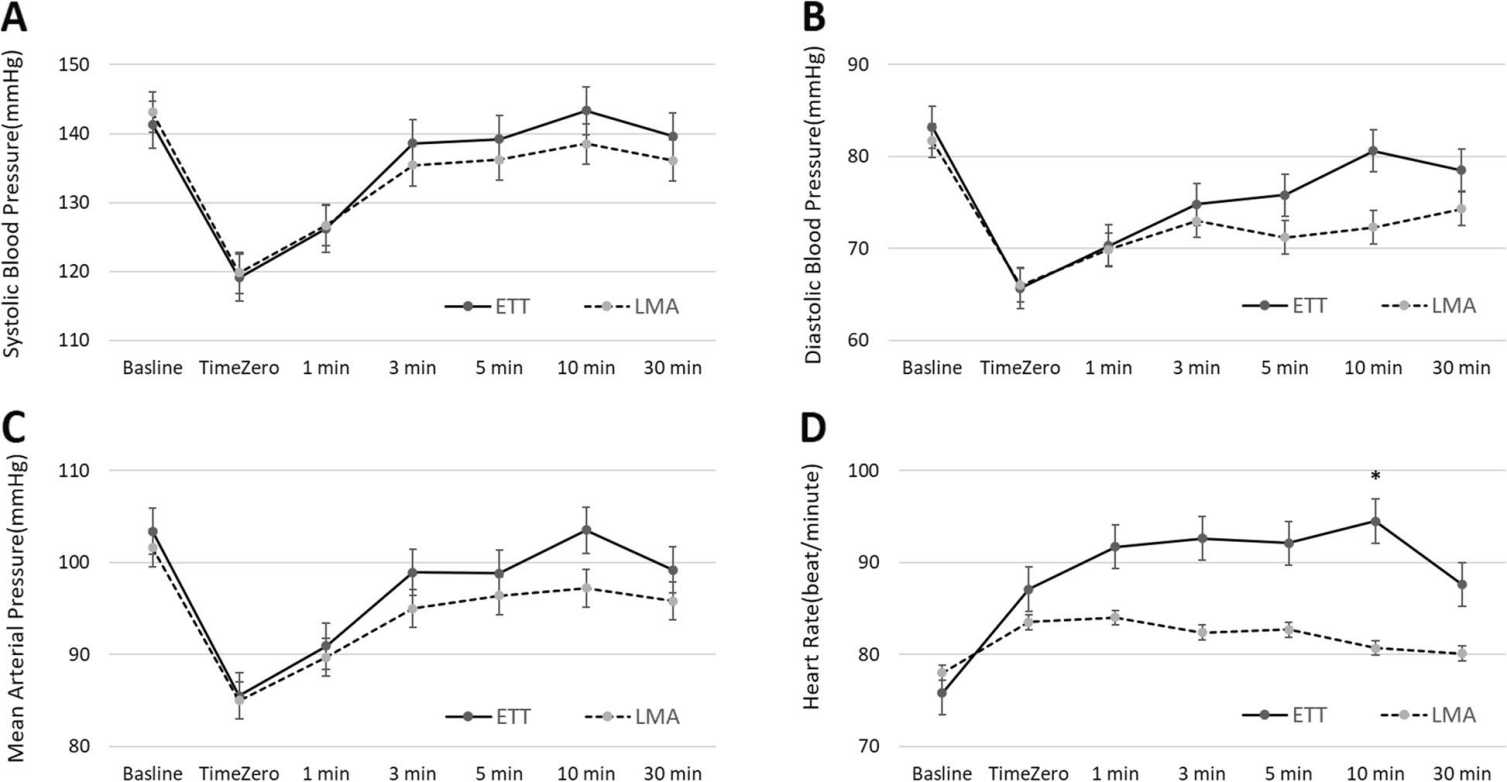
Changes in hemodynamic variables of patients in ETT Group and in LMA Group at baseline, endotracheal extubation (Time Zero), and at 1, 3, 5, 10, 30 min after Time zero. **A** Systolic blood pressure, **B** Diastolic blood pressure, **C** Mean arterial pressure, **D** Heart rate. (Bonferroni test;*p < 0.05)

From Time Zero on, blood pressures (systolic, diastolic and mean) and heart rate in ETT Group were generally higher than those in LMA Group, but the differences were insignificant at all time points except heart rate at 10 min after Time Zero, 94.5 (± 15) in ETT Group vs. 80.7 (± 15) in LMA Group (Fig. 3).

All of the secondary outcomes between the two groups were insignificantly different (Table 3).

**Table 3:** Secondary outcomes

	**ETT group (*n* = 29)**
Re-operation in 24 h	None
Stay time in intensive care unit (hours)	29.5 ± 9.4 [24 to 32.5]
Postoperative Hospital days	10.6 ± 7.0 [6 to 12.5]

Data are mean ± SD, median [IQR]. *IQR* interquartile range, difference of means (95% CI)

## Discussion

Early and smooth endotracheal extubation benefits patients undergoing craniotomy for postoperative care. No respiratory agitation and stable hemodynamics during emergence from anesthesia indicate minimal or no physical stress [1, 2], which will help maintain stable intracranial pressure [3].

Although deep extubation reduces laryngeal reflexes and cough during emergence from anesthesia [7], it could cause airway obstruction, aspiration or pulmonary edema [5]. We employed Bailey Maneuver in LMA Group at ETSev-con 2.5% [17] in order to prevent patients from coughing and airway obstruction and hemodynamic instability [12]. Moreover, the LMA could be uneventfully removed at 1% end-tidal concentrations of sevoflurane, an anesthesia level for removal of LMA suggested by Shim et al. [16]. Apart from deep extubation and Bailey Maneuver, anesthesia providers often inject intravenously lidocaine, dexmedetomidine and remifentanil to inhibit the airway response [13]. However, these drugs would introduce adverse effects to our study patients; overdose lidocaine may induce cardiac arrhythmia and neurotoxicity, remifentanil would increase the risks of postoperative nausea and vomiting and dexmedetomidine has a propensity for bradycardia and hypotension [14]. All the unfavorable effects might undermine the postoperative care for these patients.

Forty one percent of patients in ETT Group experienced mild coughs during the study period, most of which never caused rigorous head movements. The incidences of snore (78%) and oxygen desaturation (SpO_2_ < 90%) (10%) in ETT Group are similar to those reported by Koga K et al. in his deep extubation group of the study [12]. The information regarding respiratory complications and airway interventions tended to be arbitrary in our study, so we examined the arterial blood gas in order to offer objective evidences. Even with various airway interventions in ETT group, PaO_2_ in ETT group was still much lower than that in LMA group at 5 min after Time Zero, but this phenomenon did not reflect on the difference of oxygen desaturation between the two groups. The value of SpO_2_ corresponding to PaO_2_ 188.9 mmHg in ETT group is still much higher than 90%, which was not counted as oxygen desaturation. Nevertheless, the physician must be more attentive to prevent the patients in ETT group from oxygen desaturation.

There was no significant difference regarding blood pressures between the two groups through emergence from anesthesia till 30 min after Time Zero. However, heart rate rose more obviously in ETT Group, and higher enough to reach a significant difference from that in LMA Group at 10 min after Time Zero. The difference of hemodynamic changes should be explained by more airway interventions worked on the patients in ETT Group.

This study is subject to some limitations. First, this was a single blind study, and all procedures were accomplished by the same physician, so personal biases could be inherited. Second, airway interventions, including use of nasal airway, chin lift and jaw thrust, may influence the readings of arterial blood gas. Third, intracranial pressure always concerns us most in patients after brain surgeries. However, there was no intracranial pressure monitor to offer us the direct data in the study.

## Conclusions

Compared with deep extubation, a LMA as a temporary airway replacement facilitates a safe and smooth emergence from anesthesia for patients undergoing craniotomy, in terms of better oxygen saturation, fewer respiratory complications, fewer airway interventions.

## Supplementary Information

**Additional file 1.**

## Data Availability

The datasets used and/or analysed during the current study available from the corresponding author on reasonable request.

## Abbreviations

ETT: Endotracheal tube
LMA: Laryngeal mask airway
ETSev-con: End-tidal sevoflurane concentration
BMI: Body mass index
ASA: American Society of Anesthesiologists
GCS: Glasgow Coma Scale
SBP: Systolic blood pressure
DBP: Diastolic blood pressure
MAP: Mean arterial pressure
HR: Heart rate
PaCO_2_: Partial pressure of carbon dioxide
PaO_2_: Partial pressure of oxygen
EtCO_2_: End-tidal carbon dioxide
